# To Dr. Bradley

**Published:** 1800-07

**Authors:** T. Denman

**Affiliations:** Old Burlington Street


### Medical and Physical Journal.

VOL. IV.] JULY, 1800. [NO. XVII.

### To Dr. BRADLEY.

DEAR SIR,

IN a former Number of the Medical Journal, you did me the favour to insert a paper, giving an account of a fact relative to the Gloucester Militia, which went to prove, that those persons who had been infected with the Cow-pox, could not afterward be infected with the Small-pox. Since that time, there have been spread many vague reports of cases, in which it was asserted, that several persons who had been inoculated for the Cow-pox had, afterward, been actually infected with the Small-pox. Presuming that some error in the nature of the matter inoculated, or in the conduct of the operation, must have been the cause of such opposite conclusions, (if there was any foundation for the reports) I take the liberty of sending you a copy of a letter, with which I was honoured, from a nobleman of distinguished worth and respectability. His Lordship could not be mistaken in his observation of what passed when his own children were inoculated for the Cow-pox; and yet, the circumstance related, may disclose the causes of these very different opinions and assertions. Nor could his Lordship have any other motive for condescending to make this communication, but what was most humane and liberal.

When the practice of inoculating for the Small-pox was first introduced into this country, and even within my memory, many were the objections made to it, for religious, moral, and medical reasons; and the disputes which arose between the favourers and opposers of the practice, were liberally and judiciously supported. But all the objections being founded in speculation or opinion, have yielded to the authority of facts and experience, and they are now almost forgotten; though the contention taught to medical men, the necessity of great care and circumspection in every step they took. But with regard to the Cow-pox, for our whole knowledge of which we are exclusively obliged to *Dr. Jenner*, I have not heard any reason, nor seen or known any fact, that has induced me to change my opinion, or to raiſe any doubt, but that many and great advantages will be derived from the introduction of the practice of inoculating for it; the force of every argument urged, and the ſpirit of all the wit employed againſt it, lying in the ſingle word *beſtial.* I remain,

Dear Sir,

Your very humble ſervant,

T. DENMAN.

*Old Burlington Street,*

*May* 29, 1800.

---

### Lord DERBY to Dr. DENMAN.

Dear Sir,

